# Bibliometric analysis of research trends on hyperbaric oxygen therapy in stroke from 2000 to 2022

**DOI:** 10.3389/fneur.2025.1455545

**Published:** 2025-06-04

**Authors:** Mei Zhou, Xinxin Chen, Dan Li, Jiao Luo, Sihui Song, Jiuhong You, Hui Ma, Cheng Huang

**Affiliations:** ^1^Department of Rehabilitation Medicine, Center for High Altitude Medicine, West China Hospital, Sichuan University, Chengdu, Sichuan, China; ^2^Key Laboratory of Rehabilitation Medicine in Sichuan Province, West China Hospital, Sichuan University, Chengdu, Sichuan, China; ^3^School of Rehabilitation Sciences, West China School of Medicine, Sichuan University, Chengdu, Sichuan, China; ^4^West China School of Nursing, Sichuan University, Chengdu, Sichuan, China

**Keywords:** hyperbaric oxygen therapy, bibliometric analysis, HBOT, stroke, research trends

## Abstract

**Background:**

Stroke primarily results from the interruption of cerebral blood flow. Hyperbaric oxygen therapy (HBOT), a noninvasive and promising therapeutic intervention, has been widely used to treat various ischemic and hypoxic conditions. Over the past two decades, extensive research has demonstrated HBOT's efficacy in reducing cerebral infarct volume, establishing it as a viable neuroprotective strategy. Given these findings, this study employs bibliometric analysis to explore emerging trends and key research foci in HBOT applications for stroke management.

**Method:**

We performed a systematic literature search on hyperbaric oxygen therapy (HBOT) and stroke using the Web of Science Core Collection (WoSCC) database, specifically the Science Citation Index Expanded (SCI-Expanded). The search was restricted to English-language articles and reviews published between January 2000 and December 2022. Data retrieval, screening, and analysis were conducted in June 2023.

**Results:**

A total of 323 publications were identified, demonstrating annual fluctuations in research output. The United States dominated HBOT-related stroke research in both publication volume and scientific impact. Shai Efrati emerged as the most prolific author in this field, while the Sackler Faculty of Medicine at Tel Aviv University was the most influential institution. The journal Stroke published the highest number of HBOT-related stroke studies. Co-citation analysis revealed “cognitive function” as the primary research focus in HBOT applications for stroke.

**Conclusion:**

This bibliometric analysis investigated the current research landscape, trends, and emerging priorities in hyperbaric oxygen therapy (HBOT) applications for stroke. The primary research focus centered on HBOT's therapeutic potential for cognitive function enhancement and chronic-phase post-stroke treatment.

## 1 Introduction

Stroke persists as a leading cause of global mortality and adult disability, with millions of new cases and deaths reported annually. The condition results from cerebral blood flow interruption, broadly classified as ischemic or hemorrhagic (1). Pathological subtypes include ischemic stroke (IS), intracerebral hemorrhage (ICH), and subarachnoid hemorrhage (SAH) (2), with IS accounting for 62.4% of cases (3). Despite progress in prehospital emergency care and therapeutic approaches, treatment options for acute ischemic stroke (AIS) remain limited. Current stroke management continues to face significant challenges, including high incidence rates, substantial economic burdens, and poor long-term prognoses (4). Effective clinical management requires a balance between etiological interventions and neuroprotective strategies. To date, intravenous tissue plasminogen activator (IV-tPA) remains the only FDA-approved pharmacological therapy for AIS, while all neuroprotective agents tested in clinical trials have failed to demonstrate efficacy (5). Hyperbaric oxygen therapy (HBOT), a noninvasive intervention involving 100% oxygen delivery at pressures exceeding one atmosphere absolute (ATA), has emerged as a promising neuroprotective approach through multifactorial mechanisms (6, 7). Tissue hypoxia triggers ischemic cell death via pathways including excitotoxicity, oxidative/nitrative stress, inflammation, and apoptosis (8). HBOT enhances blood oxygen solubility and diffusion capacity, facilitating oxygen delivery to hypoperfused tissue regions beyond compromised vasculature (9). This mechanism suggests that elevated oxygen levels in ischemic tissues may provide neuroprotection, with HBOT estimated to increase oxygen supply to penumbral regions by 20% (10). Preclinical evidence indicates HBOT's ability to reduce oxidative stress, inflammation, and neuronal apoptosis, thereby promoting post-stroke functional recovery (11). When administered within 0–12 h of AIS onset alongside recanalization therapy, preclinical models demonstrate that HBOT reduces infarct volume, stabilizes the blood-brain barrier, and improves neurological outcomes (9). Sun et al. (12) reported that HBOT not only decreased infarct size but also reduced thrombolysis-associated parenchymal hemorrhagic complications. Furthermore, HBOT correlates with lower recurrent stroke incidence and may induce ischemic preconditioning when administered before cerebral ischemia (13–15). Regarding hemorrhagic stroke and chronic ischemic stroke, Li et al. (16) demonstrated HBOT's efficacy in improving survival and functional outcomes in surgically treated patients with acute severe hypertensive basal ganglia hemorrhage. Notably, studies emphasize HBOT's potential to induce neurological improvements even during the chronic phase of stroke (17).

Despite substantial evidence supporting hyperbaric oxygen therapy's (HBOT) efficacy in reducing infarct severity and its therapeutic potential (18), a comprehensive bibliometric analysis in this field remains lacking. Bibliometric analysis—a quantitative approach for evaluating scientific literature and mapping research trends within specific disciplines—has been widely employed to identify historical patterns and emerging research priorities (19, 20). This study presents a bibliometric analysis of HBOT applications in stroke to evaluate global research trends and identify emerging focal points from 2000 to 2022.

## 2 Methods

### 2.1 Search strategy and data collection

The publications related to HBOT and stroke were searched in the Web of Science Core Collection database (WoSCC)-Science Citation Index Expanded (SCI-Expanded) Editions. To perform a comprehensive search, the search strategy was applied as TS = ((hyperbaric oxygenation) OR (hyperbaric oxygenations) OR (hyperbaric oxygen therapy) OR (hyperbaric oxygen therapies)) AND TS = ((stroke) OR (strokes) OR (cerebrovascular accident) OR (cerebrovascular accidents) OR (CVA) OR (CVAs) OR (cerebrovascular apoplexy) OR (brain vascular accident) OR (brain vascular accidents) OR (cerebrovascular stroke) OR (cerebrovascular strokes) OR (apoplexy) OR (cerebral stroke) OR (cerebral strokes) OR (acute stroke) OR (acute strokes) OR (acute cerebrovascular accident) OR (acute cerebrovascular accidents)) from January 1, 2000, to December 31, 2022. Publication type was confined to articles and review articles published in English. Full records, including titles, authors, keywords, country, institution and references of each publication, were collected. All data were converted to plain text format by the WoS tool and then imported to CiteSpace V6.2. R4, 64-bit for further bibliometric analysis.

### 2.2 Bibliometric analysis

The analysis examined several key characteristics: annual publication trends, contributing countries and institutions, influential authors, co-cited authors, clustered networks of co-cited references, keywords, and highly cited references with citation bursts. CiteSpace, a scientometric software, was primarily employed for clustering analyses, while Microsoft Excel visualized publication trends. This analytical tool enables identification of critical developments in research fields by detecting emerging trends and patterns in scholarly literature (21). In the generated networks, node size reflects citation frequency meeting inclusion criteria, while concentric color bands indicate temporal distribution (with each hue representing a specific publication year). Burst detection analysis reveals temporal citation surges, identifying potential research hotspots within defined periods.

## 3 Results

### 3.1 Analysis of publication outputs

**Figure 1:**
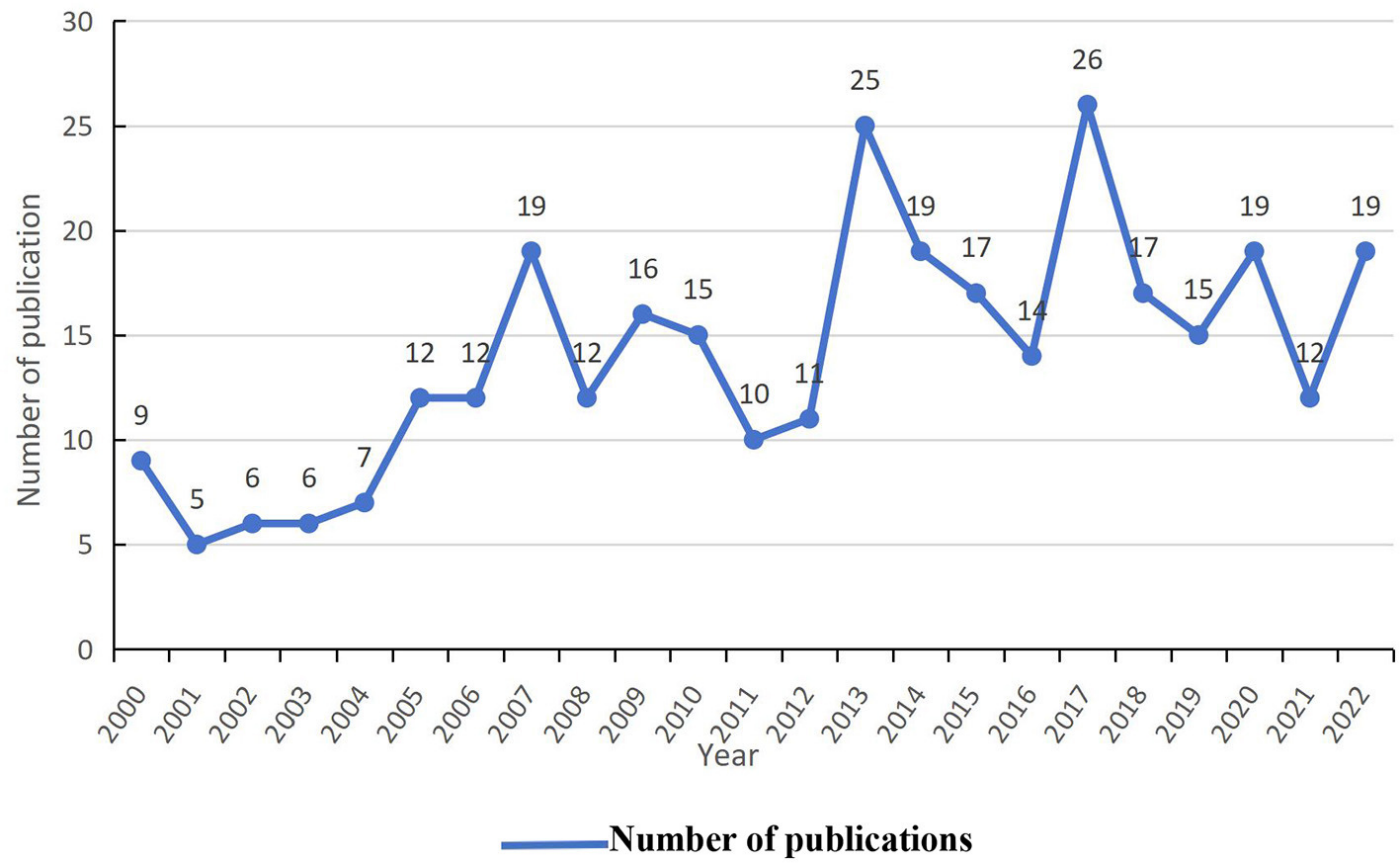
Trend of publication outputs from 2000 to 2022 on hyperbaric oxygen therapy in stroke.

The annual volume of publications serves as an indicator of a field's temporal evolution. A total of 323 original and review articles were selected for bibliometric analysis. As illustrated in Figure 1, annual publications spanning 2000–2022 ranged from 5 to 26, with the trend categorized into two distinct phases. The initial phase (2000–2004) averaged fewer than 10 publications annually. The subsequent phase (2005–2022) exhibited intermittent growth, rising to 25 publications in 2013 and peaking at 26 in 2017. The most prolific institutions were the Sackler Faculty of Medicine, Chi Mei Hospital, Tel Aviv University, Harvard University, and Capital Medical University (Table 1).

**Table 1:** Top 10 countries/regions and institutions in terms of publications for HBOT in stroke.

**Rank**	**Country/Region**	**Publications**	**Institution**	**Publications**
1	USA	110	Sackler Faculty of Medicine	16
2	Peoples R China	71	Chi Mei Hospital	15
3	Germany	29	Tel Aviv University	15
4	Taiwan	26	Harvard University	14
5	Israel	20	Capital Medical University	13
6	Canada	11	Massachusetts General Hospital	13
7	Turkey	11	Shamir Medical Center (Assaf Harofeh)	12
8	Japan	11	Ruprecht Karls University Heidelberg	10
9	South Korea	10	Loma Linda University	9
10	England	10	Leipzig University	8

### 3.2 Analysis of countries/regions

A total of 38 countries and regions contributed to the publication of 323 papers on hyperbaric oxygen therapy (HBOT) in stroke research. As shown in Table 1, the top 10 countries and regions by publication output are listed. Over a two-decade period, the United States led with the highest number of publications (*n* = 110, 34.1%), followed by China (*n* = 71, 22.0%), Germany (*n* = 29, 9.0%), Taiwan (*n* = 26, 8.0%), and Israel (*n* = 20, 6.2%). Notably, the United States alone accounted for nearly one-third of the total publications. These five regions collectively dominated the field, contributing ~80% of all HBOT-related publications on stroke.

**Figure 2:**
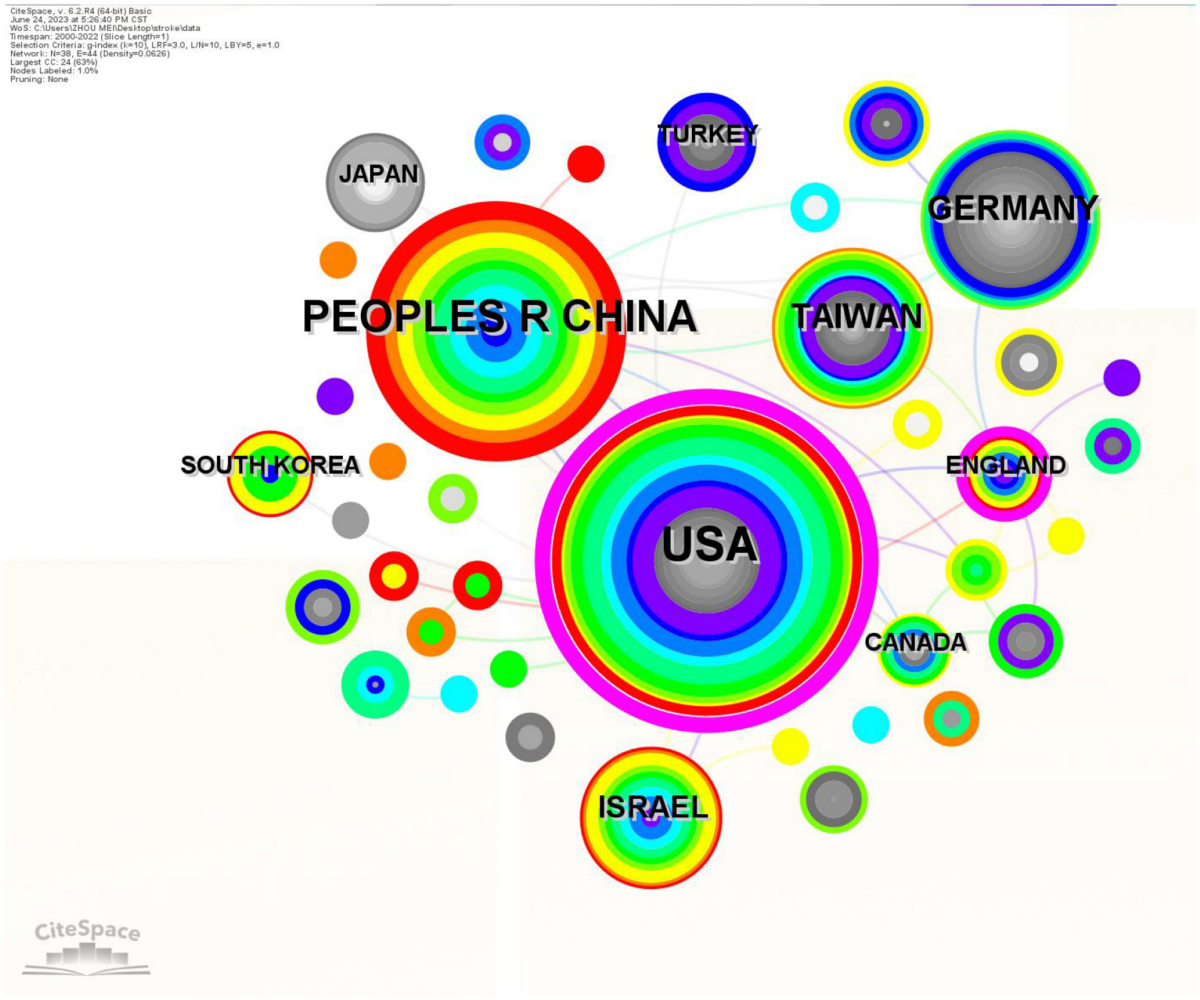
The network map of collaborating countries/region.

Figure 2 illustrates the collaboration network among countries. The thickness of the links between nodes represents the strength of cooperation between countries or regions. Notably, the United States developed extensive and close research collaborations with other countries. Additionally, eight countries and regions formed cooperative partnerships with China, England, and Germany. However, international collaboration in stroke research involving hyperbaric oxygen therapy (HBOT) remained relatively limited.

### 3.3 Analysis of authors

**Figure 3:**
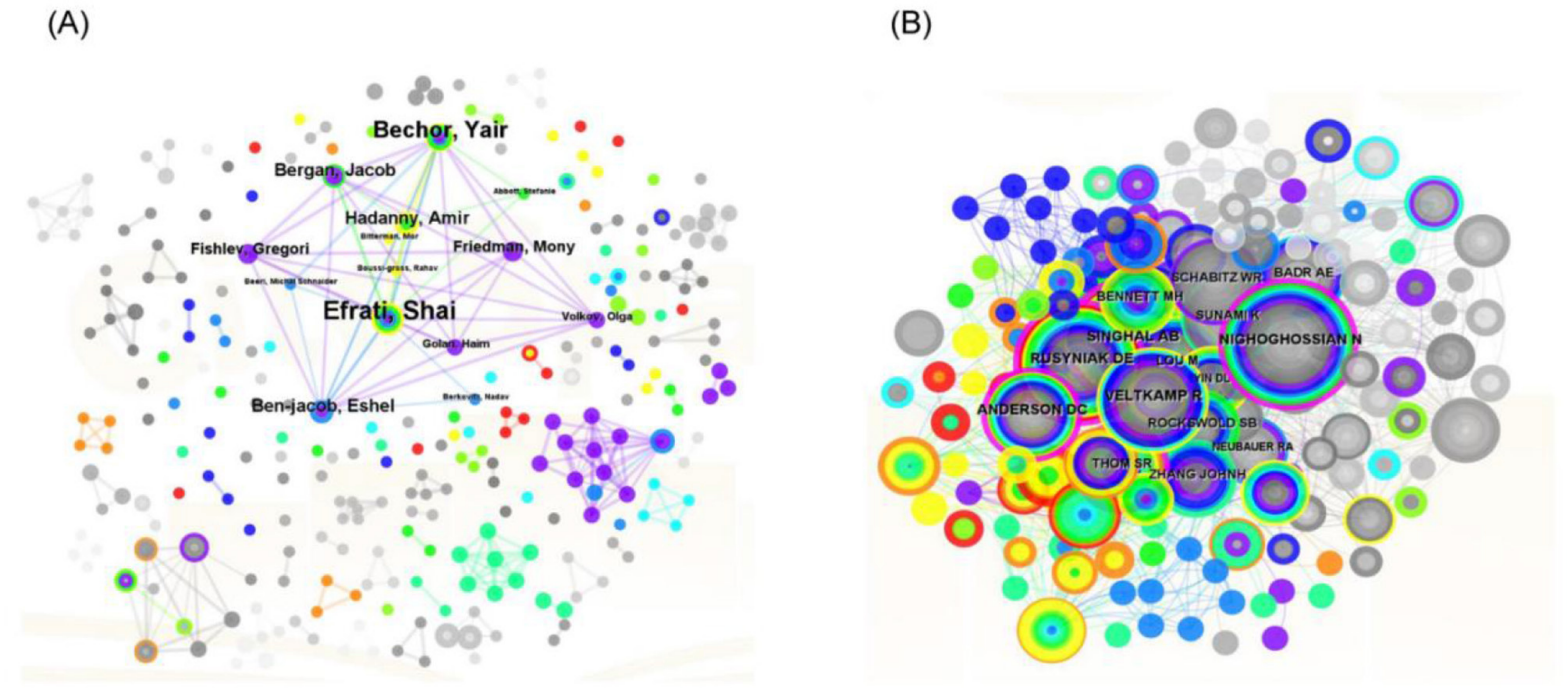
Network map of authors **(A)** and the cocited authors **(B)** on HBOT in stroke.

From 2000 to 2022, 273 authors published research on hyperbaric oxygen therapy (HBOT) for stroke. The low author density (0.0083) indicates a relatively dispersed research community. The most prolific authors were Shai Efrati (9 publications), Mao-Tsun Lin (7 publications), and Yair Benchor (7 publications). Notably, Efrati and Benchor collaborate at Israel's Assaf Harofeh Medical Center, while Lin is affiliated with Taiwan's Chi Mei Medical Center. Efrati's research primarily examines HBOT effects on brain damage, particularly in stroke and traumatic brain injury patients, with recent focus on cognitive improvement in post-stroke patients (22). Benchor similarly investigates cognitive function and neuroplasticity in stroke recovery (17, 22, 23). In contrast, Lin's work concentrates on HBOT applications for heatstroke in animal models (Figure 3).

### 3.4 Analysis of institutions

**Figure 4:**
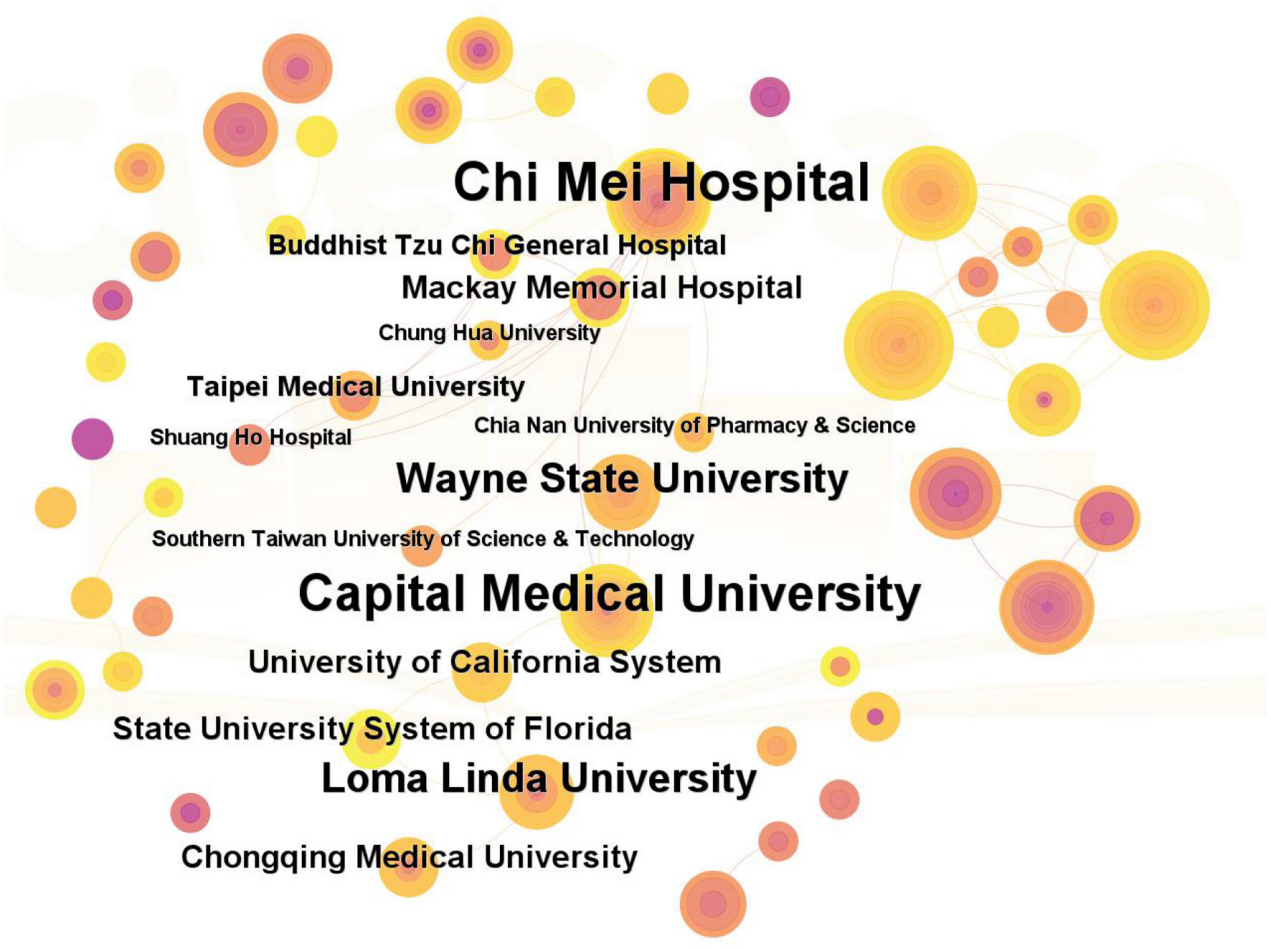
Network map of institutions on HBOT in stroke.

Figure 4 presents an institutional collaboration map for hyperbaric oxygen therapy (HBOT) in stroke. A total of 201 institutions contributed to this study. Each node represents an institution, with the size of the node proportional to the number of publications. The connections between nodes indicate collaborative relationships. The top 10 institutions are listed in Table 1. The institution with the highest number of publications was the Sackler Faculty of Medicine, followed by Tel Aviv University in Israel and Chi Mei Hospital in Taiwan.

### 3.5 Analysis of journals and cocited journals

**Figure 5:**
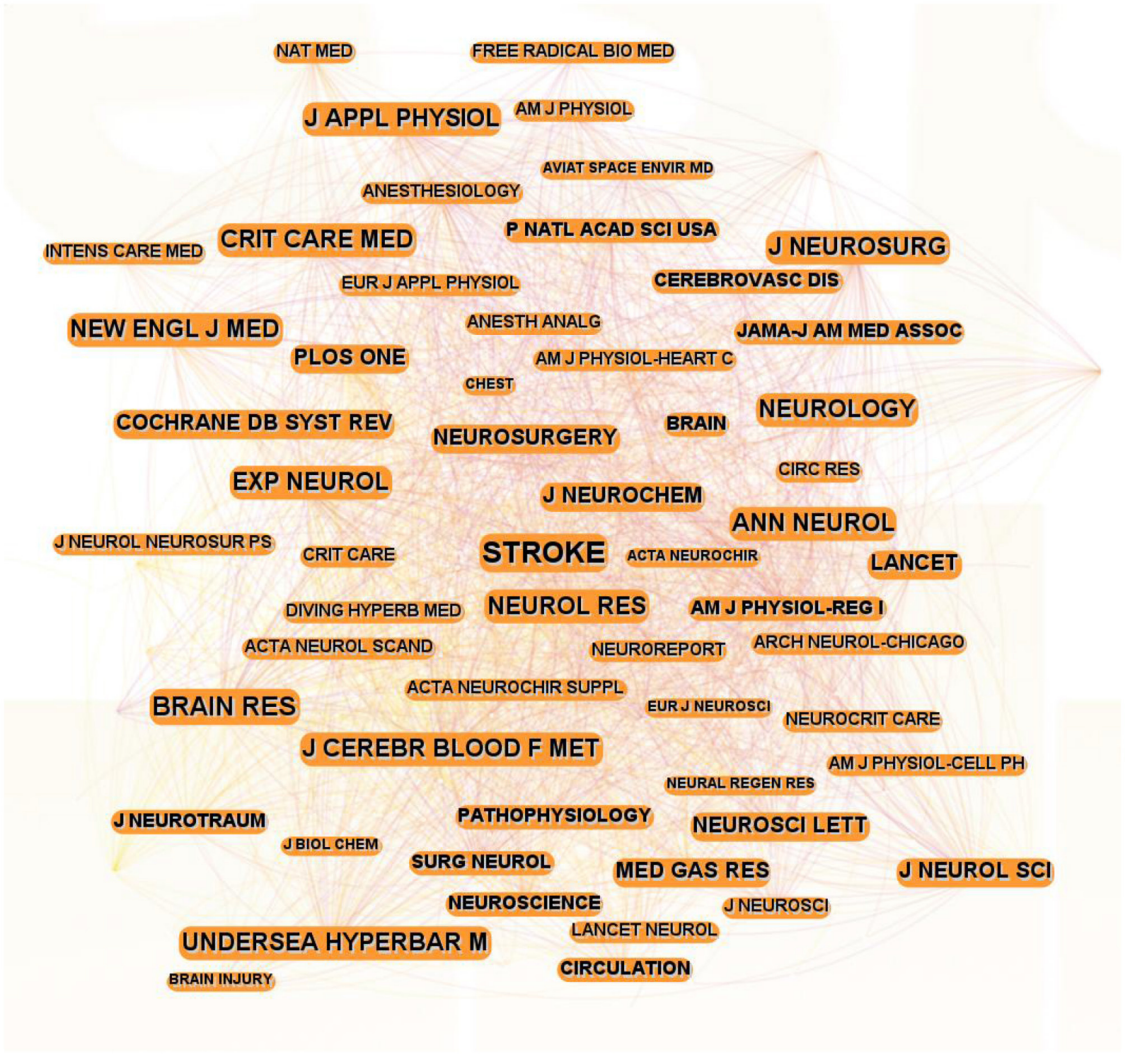
Network map of journals on HBOT in stroke.

Figure 5 presents the leading global journals that have published scientific articles on hyperbaric oxygen therapy (HBOT) in stroke research. In the overlay map, each circle represents a scientific journal, with the size of the circle reflecting the journal's citation weight. The top 10 journals, ranked by co-citation frequency, are listed in Table 2. The most frequently cited journal was Stroke (260 citations), followed by Brain Research (166 citations), Journal of Cerebral Blood Flow and Metabolism (147 citations), Critical Care Medicine (147 citations), and Neurology (118 citations). Experimental Neurology (0.16) had the highest centrality.

**Table 2:** Top 10 count journals of HBOT in stroke.

**Ranking**	**Journal**	**Count**	**Centrality**
1	Stroke	260	0.02
2	Brain Res	166	0.07
3	J Cerebr Blood F Met	147	0.1
4	Crit Care Med	119	0.08
5	Neurology	118	0.04
6	Undersea Hyperbar M	117	0.1
7	J Neuosurg	114	0.08
8	Neurol Res	109	0.07
9	New Engl J Med	105	0.04
10	Exp Neurol	100	0.16

### 3.6 Analysis of cocited references

**Figure 6:**
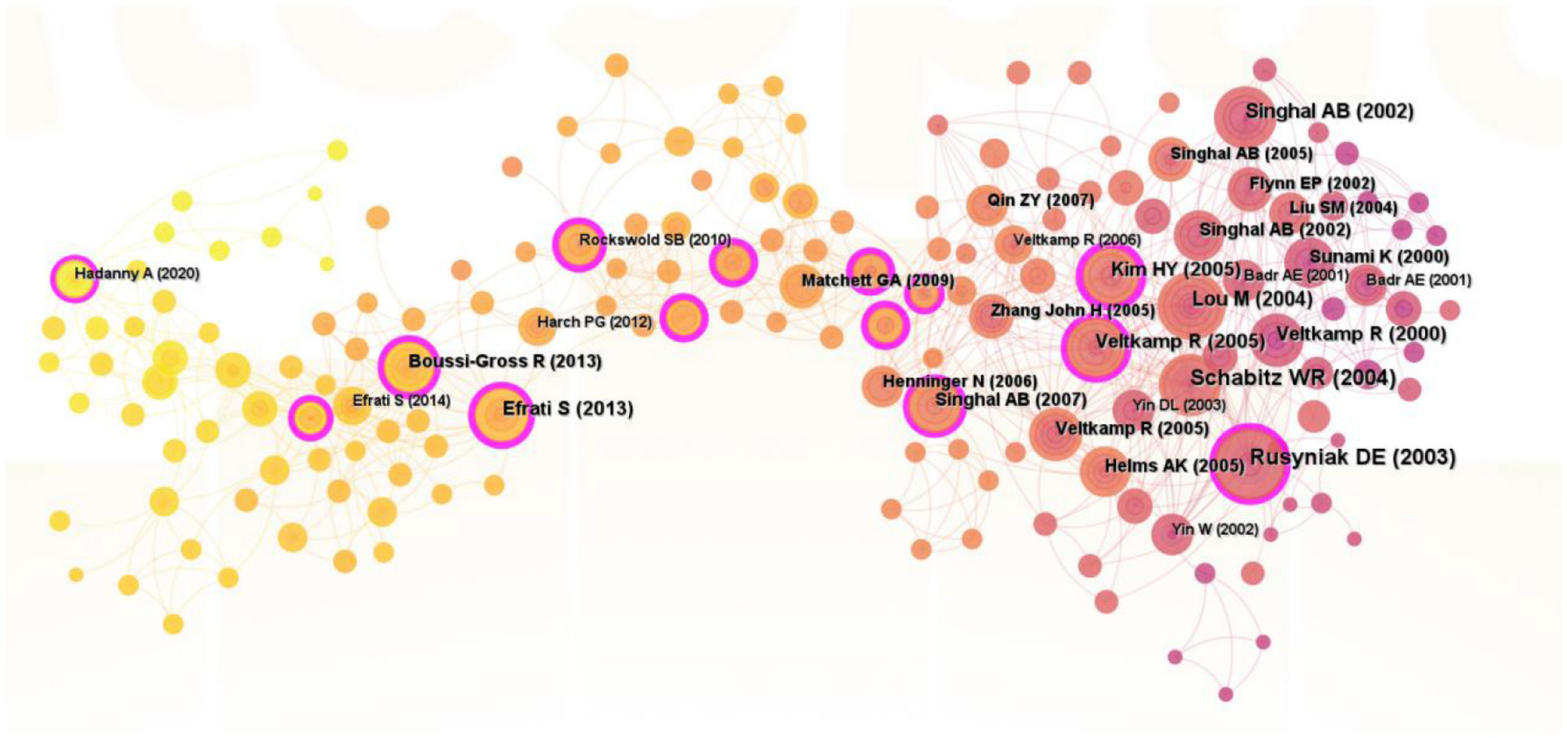
Map of cocited references of HBOT in stroke.

Figure 6 illustrates a co-citation network map of references related to hyperbaric oxygen therapy (HBOT) in stroke research. The map comprises 207 nodes, where node size corresponds to the frequency of co-cited references, and connecting lines represent co-citation relationships. Table 3 lists the top 10 co-cited references. The most frequently co-cited article, “Hyperbaric Oxygen Therapy in Acute Stroke: Results of the Hyperbaric Oxygen in Acute Ischemic Stroke Trial Pilot Study” by Rusyniak et al. (32), was published in Stroke. This was followed by studies by Schäbitz et al. (69) and Lou et al. (35). Our analysis indicates that the top 10 co-cited articles primarily focus on HBOT's effects on infarct volume, blood-brain barrier integrity, therapeutic time windows, and neuroimaging outcomes in stroke models.

**Table 3:** Top 10 cocited references related to HBOT in stroke in terms of cocitations.

**Ranking**	**Cocitation counts**	**Cited reference**	**Representative author (publication year)**
1	29	Hyperbaric oxygen therapy in acute ischemic stroke—Results of the hyperbaric oxygen in acute ischemic stroke trial pilot study	Rusyniak DE (2003)
2	26	Neuroprotection by hyperbaric oxygenation after experimental focal cerebral ischemia monitored by MRI	Schäbitz WR (2004)
3	23	Therapeutic window for use of hyperbaric oxygenation in focal transient ischemia in rats	Lou M (2004)
4	21	Normobaric hyperoxia reduces MRI diffusion abnormalities and infarct size in experimental stroke	Singhal AB (2002)
5	20	Hyperbaric oxygen reduces blood–brain barrier damage and edema after transient focal cerebral ischemia	Veltkamp R (2005)
6	19	Normobaric hyperoxia extends the reperfusion window in focal cerebral ischemia	Kim HY (2005)
7	16	Hyperbaric Oxygen Induces Late Neuroplasticity in Post Stroke Patients—Randomized, Prospective Trial	Efrati S (2013)
8	16	Hyperbaric oxygen decreases infarct size and behavioral deficit after transient focal cerebral ischemia in rats	Veltkamp R (2000)
9	15	A review of oxygen therapy in ischemic stroke	Singhal AB (2007)
10	15	Hyperbaric oxygen reduces infarct volume in rats by increasing oxygen supply to the ischemic periphery	Sunami K (2000)

**Figure 7:**
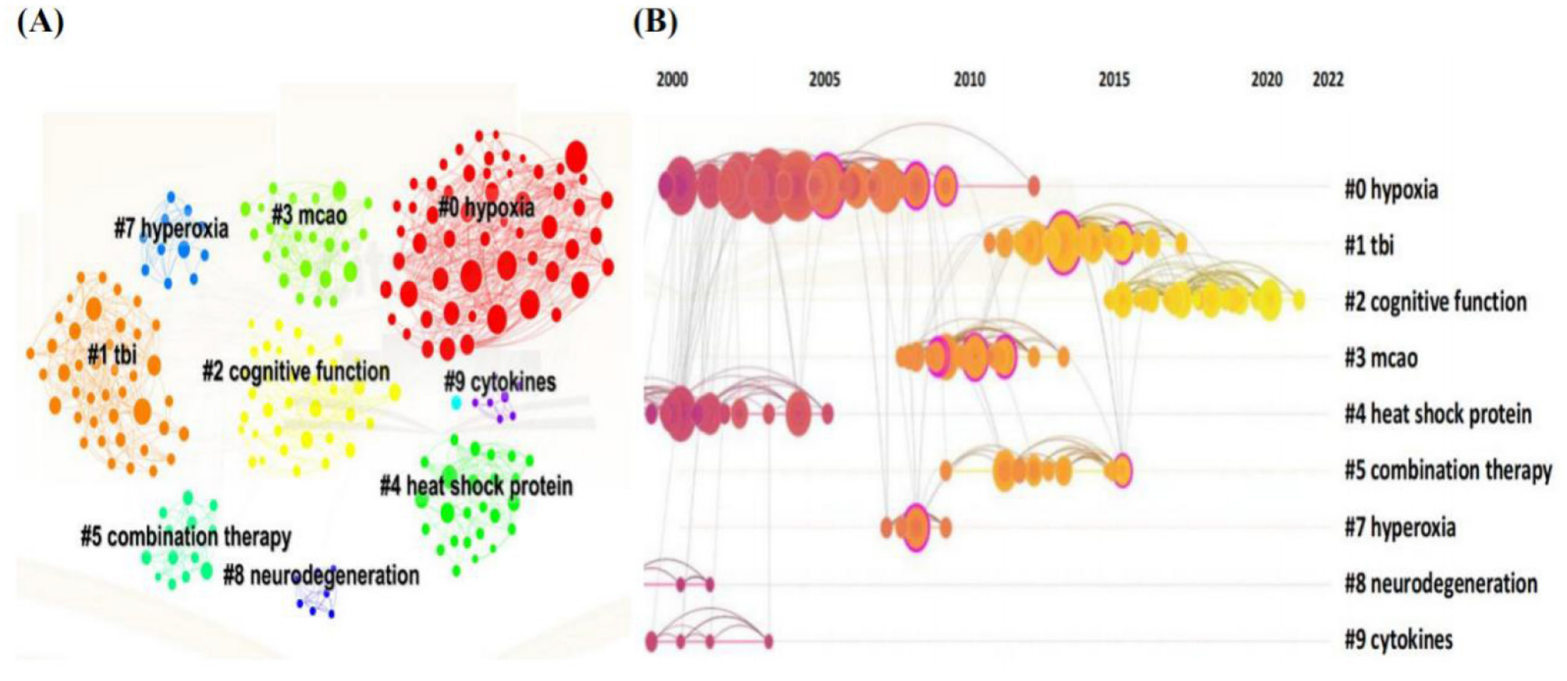
Clustered network map of cocited references **(A)** with their clustered labels on the right **(B)** on HBOT in stroke.

Co-citation clustering is a valuable method for identifying emerging research frontiers (24). Subsequent analysis of these co-cited references yielded 299 nodes, 1,085 edges, and 52 distinct clusters (Figure 7A), accompanied by timeline visualizations for each cluster label (Figure 7B). The spatial distribution of nodes within these clusters reveals that early HBOT research primarily focused on traumatic brain injury, cognitive function, and the combination of HBOT with normobaric hyperoxia therapy or other adjunctive treatments.

### 3.7 Analysis of keywords and reference citation burst

The burst keyword represents emerging concepts that have been frequently cited over a specific period. Over the past two decades, hyperbaric oxygen therapy ranked first with the highest burst strength (5.79), followed by oxidative stress (3.83) and traumatic brain injury (3.8). In the last 5 years (2018–2022), the most prominent keywords were oxidative stress, hyperbaric oxygen therapy, ischemic stroke, and Alzheimer's disease.

Reference citation burst detection typically identifies works of significant potential or interest, highlighting key areas within the academic field (25). The paper by Rusyniak, published in Stroke, ranked first with the highest burst strength (10.6) (26), also recording the highest citation numbers mentioned above. Three additional papers—Hadanny A, Restore Neurol Neuros (4.83), Tal S, Front Hum Neurosci (4.38), and Rosario ER, Neurol Res Int (3.75)—emerged as bursts until 2022. These papers established physical impairments, neurocognitive functions, mechanisms, neuroplasticity, and traumatic brain injury as key research hotspots in HBOT.

## 4 Discussion

### 4.1 Primary findings

This study employed bibliometric analysis to examine 323 publications pertaining to hyperbaric oxygen therapy (HBOT) in stroke from 2000 to 2022. The United States emerged as the leading contributor to this research domain, with Tel Aviv University's Sackler Faculty of Medicine producing the highest number of publications. The journals Stroke, Brain Research, and the Journal of Cerebral Blood Flow and Metabolism were identified as the most prolific and impactful in this field. Cluster analysis of co-cited publications revealed that studies over the past two decades primarily focused on combination therapy, traumatic brain injury, and cognitive function. Furthermore, reference citation burst analysis highlighted cognitive function as a prominent research hotspot in the application of HBOT for stroke.

### 4.2 Results of the study in context

#### 4.2.1 The current status of HBOT research in stroke

Over the past 5 years, annual publication numbers fluctuated below 20, reflecting diminished research activity in hyperbaric oxygen therapy (HBOT) for stroke. Mijajlovic et al. has noted a notable decline in interest, potentially attributable to conflicting and complex evidence regarding HBOT efficacy in acute ischemic stroke (AIS) (27). HBOT application in ischemic stroke models involves three primary phases: pretreatment prior to stroke onset, early intervention in AIS, and neurological recovery during the chronic phase. Numerous preclinical studies have demonstrated that HBOT reduces infarct volume, minimizes hemorrhagic risk, and enhances functional recovery post-ischemia (26). However, no sufficiently powered, rigorously controlled clinical trials have conclusively evaluated HBOT efficacy in AIS (28). Per the 2016 Tenth European Consensus Conference on Hyperbaric Medicine, HBOT is not recommended for acute ischemic stroke (Type 1 recommendation, Level C evidence) (29). Three clinical trials failed to demonstrate clinical benefits, though methodological limitations—including small cohorts, suboptimal control groups, and delayed HBOT initiation—may explain these outcomes (30–32). For instance, Anderson et al. enrolled patients up to 2 weeks post-stroke, potentially exceeding the therapeutic window. Veltkamp et al. (33) emphasized that HBOT improves outcomes only when administered early after transient focal ischemia, a finding corroborated by Zhang et al., who reported neuroprotection at 3–6 h post-stroke but detrimental effects at 12–23 h (34). Subsequent studies confirmed that HBOT initiated within 6 h reduces neuronal damage, whereas delayed treatment exacerbates functional and histological deficits (35). These findings underscore the critical importance of patient selection and HBOT timing. In a 2003 Stroke study by Rusyniak et al., the HBOT group received 100% oxygen at 2.5 ATA for 60 minutes, while the sham group was administered 100% oxygen at 1.14 ATA. We aim to highlight that normobaric oxygen therapy (the sham group) itself may exert neuroprotective effects during early ischemia, which has led some researchers to question the validity of this sham control design. For instance, Kim et al. (36) and Zhang et al. (37) have raised such concerns. A key translational challenge lies in discrepancies between preclinical and clinical study designs (38). Methodological variability arises from divergent stroke models (global, permanent, or transient ischemia), species (gerbils, dogs, cats, rabbits, and rats), inadequate sample sizes, suboptimal sham controls, inconsistent blinding protocols, treatment timing, and precise HBOT parameters (pressure, dose, regimen). Advancements in diagnostic technologies, such as transcranial Doppler or MR/CT angiography for recanalization monitoring, could refine trial design (28). At the cellular level, neuroimaging modalities can delineate tissue damage, while electrophysiological assessments may detect ischemic insults, exemplified by anoxic terminal negativity (39, 40). Integrating advanced neuroimaging with rigorous methodology, future research should prioritize evidence-based, randomized, blinded trials to clarify HBOT's role in stroke management.

A further consideration involves HBOT's influence on cerebrovascular pathophysiology. The physiological mechanisms of HBOT—including hyperoxygenation, vasoconstriction, and angiogenesis—exhibit region-specific effects (27). Nakajima et al. (41) demonstrated that oxygen-induced vasoconstriction occurs in healthy cerebral vasculature but not in ischemic regions, where cerebral blood flow paradoxically increases.

#### 4.2.2 Active cooperation is necessary for HBOT in stroke research

Among the top 10 countries/regions, two were developing nations (Turkey and China), while the majority of the remaining eight were developed economies in Asia, Europe, and North America, collectively representing 70% of the total. The United States emerged as the foremost contributor in both publication volume and centrality metrics. Notably, 60% of the top 10 institutions and highly cited authors were affiliated with Israel and the United States. These findings indicate that the United States and Israel hold dominant positions in this field, with their researchers and institutions serving as central contributors to advancements in the area.

By analyzing the publications and citations, Sackler Faculty of Medicine, Chi Mei Hospital, and Tel Aviv University were the most impactful and prolific institutions. To some extent, it indicated that these institutions were the main research forces and had a relatively high quality of published papers in this domain. Efrati Shai, Lin Mao-Tsun, and Bechor Yair were the most active and influential authors. Efrati Shai and Bechor Yair were from Israel, while Lin Mao-Tsun was from Taiwan. As we can see in Figure 3, stable and extensive cooperation between top authors was established in the network map of authors, especially among high-yield authors (Efrati Shai, Bechor Yair, Ben-jacob Eshel, and Hadanny Amir). However, the scattered lines between other authors indicated that there was poor cooperation among authors in terms of HBOT in stroke. Additionally, few connections between countries implied that there were weak cooperative relationships with countries on HBOT in the stroke academic field (Figure 2). Overall, these findings suggested that more scientific collaborations among authors and national/regional cooperation between countries/regions were needed.

#### 4.2.3 The major trends and hot issues of HBOT in stroke

**Figure 8:**
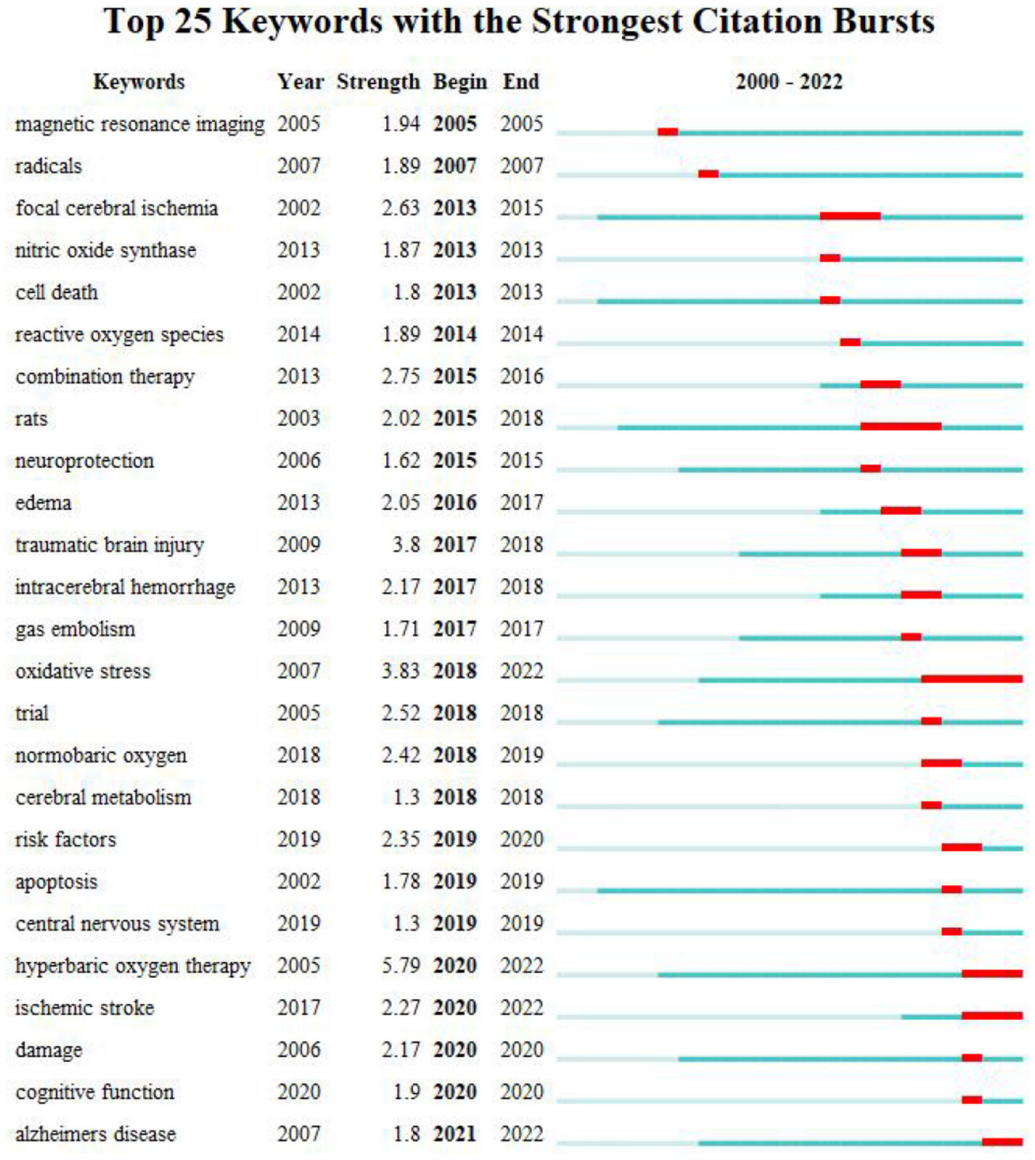
Keywords with the strongest citation bursts in publications on HBOT for stroke between 2000 and 2021.

**Figure 9:**
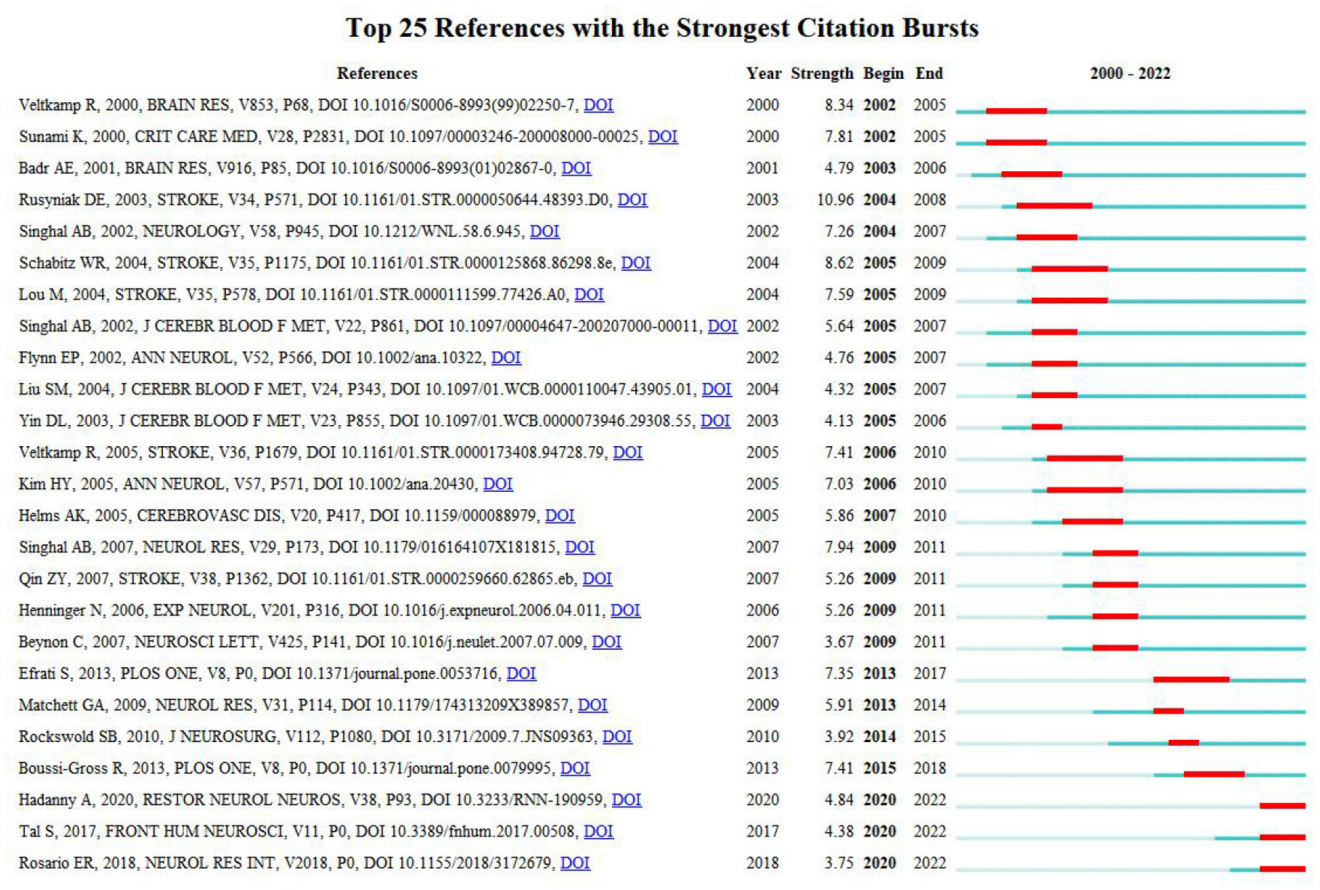
References with the strongest citation burst in publications on HBOT in stroke between 2000 and 2022.

Burst keywords have enabled researchers to quickly identify emerging areas of research within a specific field and provide guidance for future studies. Over the past 5 years (2018–2022), the most significant hotspots included “oxidative stress”, “hyperbaric oxygen therapy”, “ischemic stroke”, and “Alzheimer's disease”. Additionally, “cognitive function” emerged as one of the key clusters following the co-citation analysis of publications. Furthermore, two of the three burst-till-now papers (22, 42, 43) (Figures 8, 9) addressed cognitive functions, particularly in post-stroke patients in the late chronic stage, while the third focused on the mechanisms of HBOT in traumatic brain injury patients. Thus, cognitive function represents a current hotspot in HBOT-related stroke research.

Although hyperbaric oxygen therapy (HBOT) is not yet FDA-approved for treating brain-related disorders, numerous studies have demonstrated enhanced cognitive performance following its application in cases of brain injury (44). The terms “chronic penumbra” and “stunned brain” refer to regions with critically reduced cerebral blood flow (CBF), abolished synaptic activity, and preserved structural integrity (45). These regions, which are damaged but not necrotic after stroke, hold therapeutic potential, as proper intervention may restore their function (46). Advanced imaging techniques further reveal that stunned brain tissue can persist for months to years following an acute ischemic event (47). Furthermore, SPECT imaging reveals that hyperbaric oxygen therapy (HBOT) induces the reactivation of neuronal activity in metabolically stunned brain regions (17). Research by Efrati et al. (17) demonstrates that repeated HBOT sessions significantly enhance neuroplasticity in regions of viable but hypoactive neurons, driving neurological improvements in patients with late chronic-stage stroke. These findings challenge the notion of a limited post-stroke recovery window, supporting the hypothesis that neuroplasticity can be reactivated months to years after the acute event through targeted neuromodulation therapies like HBOT. Consistent with this, Hadanny et al. (22) report that HBOT promotes significant improvements in global cognitive function during the late chronic stage. Mechanistically, these effects are linked to cerebral angiogenesis, increased cerebral blood flow (CBF) and brain volume, and enhanced microstructural integrity of cerebral white and gray matter (43). In addition, Golan et al. (48) pioneered the use of single-photon emission computed tomography (SPECT) imaging as a diagnostic tool to monitor therapeutic outcomes of hyperbaric oxygen therapy (HBOT) in post-stroke patients. SPECT/CT-based quantification of penumbral volume may become an effective method for assessing HBOT efficacy in individuals with ischemic stroke. Furthermore, advanced neuroimaging modalities, such as functional magnetic resonance imaging (fMRI), quantitative electroencephalography (qEEG), and functional near-infrared spectroscopy (fNIRS), have been integrated into HBOT research. For instance, Cevolani et al. (49) utilized fMRI to evaluate HBOT-induced functional changes in the central nervous systems of stroke patients, demonstrating its sensitivity in detecting neural activity before and after treatment. Given these advancements, cognitive function is poised to emerge as a key focus in future studies. Investigations combining neuroimaging with assessments of neurocognitive recovery in chronic-phase stroke patients—particularly to elucidate HBOT's mechanistic pathways—are likely to dominate upcoming research efforts.

### 4.3 The main mechanisms of hyperbaric oxygen in stroke

Hyperbaric oxygen therapy (HBOT) increases dissolved oxygen content in plasma and extends oxygen diffusion distance, enabling rapid oxygen delivery to ischemic regions and improving tissue oxygenation to preserve neurological function. The “normobaric oxygen paradox” or “hyperoxia-hypoxia paradox” plays a crucial role in HBOT's therapeutic effects (50, 51). During HBOT, oxygen levels increase from 21 to 100%, then return to 21% post-treatment. This paradox describes how cells detect oxygen fluctuations through specific chemoreceptors, triggering hypoxia-induced cellular cascades that enable intermittent hyperoxia to promote tissue regeneration without hypoxia-related damage (51). Elevated oxygen concentrations induce various mediators and cellular matrices required for regeneration during hypoxia, including upregulation of oxygen-sensitive genes and activation of cellular repair processes (52, 53).

Research indicates HBOT exerts neuroprotective effects through multiple pathways. The primary mechanisms by which HBOT improves stroke outcomes include: (1) stimulating expression of neurotrophic factors in ischemic stroke (IS) rats, promoting bone marrow mesenchymal stem cell homing to ischemic brain regions and enhancing cellular repair (54); (2) increasing antioxidant enzymes and glutathione metabolism while reducing oxidative stress, thereby decreasing cerebral infarction volume after ischemia-reperfusion injury (55); (3) inhibiting neutrophil infiltration into infarcted areas, attenuating blood-brain barrier damage, and reducing inflammatory cytokines to mitigate neuroinflammation (56); (4) downregulating hypoxia-inducible factor-1 (HIF-1) and its downstream effector proteins to suppress neuronal apoptosis and inflammatory responses (57).

### 4.4 The efficacy of HBOT in different diseases

The application of HBOT in ischemic stroke can be categorized into three phases: pretreatment before onset, acute ischemic stroke (AIS) early phase, and AIS chronic phase. The early phase is further divided into the acute phase (0–24 h) and subacute phase (1–5 days) (58). Current evidence confirms HBOT's safety and efficacy during the subacute and chronic phases of stroke (22, 59), though insufficient data exist to demonstrate significant benefits in the acute phase (60). This discrepancy primarily stems from inconsistencies between animal studies and clinical trial outcomes. While animal models support HBOT's effectiveness when administered within 12 h of stroke onset (28), the narrow therapeutic window for AIS severely limits treatment options beyond thrombolysis or thrombectomy.

Post-stroke cognitive impairment represents one of the most common stroke complications. Research demonstrates HBOT's capacity to improve motor function and memory in chronic stroke patients. Tal et al. (43) reported HBOT's ability to induce neuroplasticity even years after brain injury. Hadanny et al. (22) further observed clinically significant cognitive improvements in 86% of patients receiving HBOT at a median of 1.5 ± 3.3 years post-stroke, defined as ≥7.5-point absolute increase in standardized cognitive domain scores. These findings suggest HBOT can significantly enhance neurological and cognitive function even during late chronic stages. Moreover, Hadanny et al.'s (61) retrospective analysis of traumatic brain injury patients with chronic cognitive impairment showed HBOT improved all cognitive domains, including memory, attention, and executive function. Notably, HBOT enhanced hippocampal recovery and overall cognitive function even when initiated 50 days post-injury (62).

### 4.5 Current status and prospect of HBOT

The prognosis of traumatic brain injury (TBI) patients is primarily determined by the initial injury severity. While ~80–90% of mild TBI patients achieve complete recovery within 2 weeks, severe TBI cases are associated with mortality rates as high as 35% (63). In recent years, HBOT has emerged as a significant therapeutic intervention for consciousness restoration, demonstrating notable efficacy in patients with prolonged coma following severe TBI (64). Disorders of consciousness present substantial rehabilitation challenges and impose significant socioeconomic burdens on both healthcare systems and affected families. Future directions in TBI may focus on: improving functional outcomes in moderate-to-severe cases, developing effective consciousness restoration approaches and advancing mechanistic understanding of TBI. Precise clinical outcome assessments would enable families to make informed treatment decisions and help healthcare institutions optimize resource allocation. Current macroscopic imaging modalities (CT/MRI) may be insufficient for comprehensive evaluation; thus, future diagnostic paradigms should integrate multimodal monitoring systems combining clinical presentation, advanced neuroimaging, functional imaging, and multiple biofluid biomarkers (e.g., blood, cerebrospinal fluid). Such integrated approaches could elucidate disease mechanisms, establish efficacy evaluation models, and provide scientific foundations for clinical treatment and prognostic assessment.

Hyperbaric oxygen preconditioning (HBO-PC) refers to preemptive hyperbaric oxygen exposure prior to critical events for preventive neuroprotection (65). In a recent study, Wang et al. (66) administered five consecutive days of HBO-PC before middle cerebral artery occlusion (MCAO) modeling in rats, demonstrating significant reductions in cerebral infarction volume, decreased oxidative stress, and improved neurological function. Dong et al. (67) investigated the efficacy of combined HBOT with butylphthalide and oxiracetam for post-stroke vascular cognitive impairment, reporting robust therapeutic effects with good safety profiles. Wei et al. (68) conducted a single-center, double-blind randomized controlled trial evaluating HBOT combined with repetitive transcranial magnetic stimulation (rTMS) for vascular cognitive impairment, enrolling 72 participants randomized into four groups: control, HBOT alone, rTMS alone, and combined therapy. The 4-week treatment period with 3-week follow-up employed comprehensive assessments including MoCA, MMSE, P300 event-related potentials, and task-state functional near-infrared spectroscopy (fNIRS). P300 potentials objectively reflect electrophysiological changes during cognitive processing, while fNIRS monitors cortical hemodynamic alterations. Given HBO-PC's demonstrated efficacy, developing standardized protocols for high-risk stroke populations may help prevent initial or recurrent strokes. For post-stroke or post-TBI cognitive impairment, future multimodal approaches can integrate: pharmacological interventions, cognitive training, aerobic exercise, nutritional strategies, non-invasive brain stimulation, and stem cell therapies. Advanced neuroimaging techniques including functional MRI, magnetic resonance diffusion tensor imaging, EEG, neurophysiological monitoring, and fNIRS should be incorporated into clinical trial designs for comprehensive treatment evaluation.

## 5 Strengths and limitations

To our knowledge, this bibliometric study represents the first systematic effort to delineate research trends and emerging hotspots in hyperbaric oxygen therapy (HBOT) applications for stroke. By analyzing 323 original research and review articles spanning two decades, this work provides a comprehensive overview of the field, highlighting key areas that may guide future investigative priorities.

This study has several limitations. First, our analysis was restricted to publications indexed in the Web of Science Core Collection (WoSCC) database, potentially excluding relevant studies from other databases such as MEDLINE, Scopus, and the Cochrane Library. Second, the inclusion of only English-language publications may have introduced selection bias, limiting the representativeness of the literature. Third, by focusing solely on peer-reviewed articles and reviews, our methodology likely omitted valuable insights from gray literature sources, including non-indexed journals, dissertations, books, and governmental reports.

## 6 Conclusion

This bibliometric analysis offers a comprehensive overview of the current status and global trends in hyperbaric oxygen therapy (HBOT) applications for stroke. The journals *Stroke, Brain Research, Journal of Cerebral Blood Flow and Metabolism, Critical Care Medicine*, and *Experimental Neurology* emerged as the most influential publications in this domain. The most prolific and impactful authors included Efrati Shai., Lin Mao-Tsun., Bechor Yair, Lo EH and Singhal AB. The Sackler Faculty of Medicine produced HBOT-related studies with the highest citation counts, underscoring its academic leadership. Geographically, research contributions were dominated by institutions in Asia, Europe, and North America, which collectively accounted for the majority of publications and highly cited articles. However, enhanced collaboration among authors, institutions, and international regions is warranted to advance the field. Current research priorities center on elucidating HBOT's effects on cognitive recovery in stroke patients, reflecting its therapeutic potential in neurorehabilitation.

## Data Availability

The original contributions presented in the study are included in the article/supplementary material, further inquiries can be directed to the corresponding author.
